# Trophic ecology of the armadillo ant,*Tatuidris tatusia*, assessed by stable isotopes and behavioral observations

**DOI:** 10.1093/jis/14.1.108

**Published:** 2014-08-12

**Authors:** Justine Jacquemin, Thibaut Delsinne, Mark Maraun, Maurice Leponce

**Affiliations:** 1 Biodiversity Monitoring and Assessment, Royal Belgian Institute of Natural Sciences, Rue Vautier 29, B-1000 Brussels, Belgium; 2 Evolutionary Biology & Ecology, Université Libre de Bruxelles, Belgium; 3 J.F. Blumenbach Institute of Zoology and Anthropology, Animal Ecology, Georg August University of Gottingen, Germany

**Keywords:** food web, Formicidae, Hymenoptera, predation, trophic biology

## Abstract

Ants of the genus *Tatuidris* Brown and Kempf (Formicidae: Agroecomyrmecinae) generally occur at low abundances in forests of Central and South America. Their morphological peculiarities, such as mandibular brushes, are presumably linked with specialized predatory habits. Our aims were to (1) assess the *Tatuidris* abundance in an evergreen premontane forest of Ecuador; (2) detail morphological characteristics and feeding behavior of *Tatuidris*; and (3) define the position of *Tatuidris* in the food web. A total of 465 litter samples were collected. For the first time, live *Tatuidris* individuals were observed. Various potential food sources were offered to them. A nitrogen stable isotope ratio analysis (^15^N/^14^N) was conducted on *Tatuidris tatusia*, other ants, and common organisms from the leaf-litter mesofauna. We found a relatively high abundance of *T. tatusia* in the site. Live individuals did not feed on any of the food sources offered, as usually observed with diet specialist ants. The isotope analysis revealed that *T. tatusia* is one of the top predators of the leaf-litter food web.

## Introduction

Ants of the genus *Tatuidris* Brown and Kempf (Formicidae: Agroecomyrmecinae) are rare inhabitants of soil and leaf-litter layers of Neotropical forests from Mexico to French Guiana, central Brazil, and Peru (Donoso 2012; Lacau et al. 2012). In his recent revision of the genus, Donoso (2012) considers the genus *Tatuidris* as monotypic, and he synonymized the recently described *T. kapasi* Lacau and Groc, 2012, from French Guiana, under *T. tatusia* (first described by Brown and Kempf, 1968).

*T. tatusia* possesses a series of morphologicalpeculiarities, such as modified mandibles, suggesting that *Tatuidris* are specialist predators (Brown and Kempf 1968). Nevertheless, their feeding habits and trophic position remain unknown. It is very difficult to find and observe these ants, but techniques such as DNA analysis of gut content or stable isotope analysis is of particular value for such an analysis because it makes it possible to define the trophic position of an organism in a food web and that organism’s degree of omnivory.

The measurement of the heavy to light isotope ratio (15N/14N) in an animal’s tissue provides information on its diet and trophic position (DeNiro and Epstein 1981; Minagawa and Wada 1984). Indeed, the N isotopic signature of a consumer is typically enriched by ≈3.4‰ relative to its diet (Post 2002; Maraun et al. 2011). Hence, the higher the position of an animal in the trophic chain, the higher the abundance of nitrogen stable isotope in its tissue. Primary consumers have low signatures, and top predators the highest ones. The degree of omnivory is reflected by the intraspecific variability of the isotopic signature (Tillberg and Breed 2004).

Stable isotopes have already been successfully used for assessing the trophic ecology of ants(Blüthgen et al. 2003; Feldhaar et al. 2009), their degree of omnivory (Tillberg and Breed 2004; Jacquemin et al. 2012), and the change in their dietary habits across habitats (Gibb and Cunningham 2011) or between their native and introduced ranges (Tillberg et al. 2007). Stable isotopes also provided information on the position of ants in food webs, relative to other ants and other taxa (Tillberg et al. 2006; Hyodo et al. 2010; Jacquemin et al. 2012).

In the current study, our aims were to (1) assess *Tatuidris* species abundance in an evergreen premontane forest of Ecuador; (2) detail its morphological characteristics, behavior, and dietary habits through a feeding experiment on a live colony; and (3) define its position in the food web using an isotopic approach.

## Materials and Methods

### Study site

The study was conducted in an evergreen premontane forest located in Copalinga Private Reserve (4.0912° S, 78.9607° W), on the eastern slope of the Ecuadorian Andes, 1000 m above sea level. High precipitation occurs from February to June, while from August to December it is drier (average annual rainfall: 2000 mm ± 387 SD; average annual temperature: 22.3°C ± 0.9 SD; C. Vits, Copalinga private reserve, personal communication, period: 2003–2011). Soil is sandy clay loam (proportion of sand, silt and clay is 43%, 20%, and 37%, respectively) with mean pH = 3.6 (± 0.2 SD, n = 100 soil samples).

### Species abundance

The calculation of species abundance was based on 220 Winkler extractions performed in November 2009 (dry season) and 245 in March 2010 (early rainy season) in Copalinga.

### Morphology

High-resolution digital photographs of *Tatuidris tatusia* habitus, mandibles, sting, and setae on the protibia are presented, along with scanning electron micrographs (SEM) for mandibles and setae. High-resolution digital images were taken using a Leica DFC290 camera attached to a Leica Z6 APO stereomicroscope (www.leica-microsystems.com). Series of images were taken by focusing the sharpness on different levels of the structure using the Leica Application Suite v38 (2003– 2011), and combined with the “Align and balance used frame (quick)” and “Do stack” commands of CombineZP (Hadley 2010). Final editing of images was done in Adobe Photoshop CS5 (www.adobe.com). SEM photographs of gold-coated specimens were taken using an FEI Quanta 200 (www.fei.com) scanning electron microscope.

Voucher specimens of *Tatuidris tatusia* were deposited at the Royal Belgian Institute of Natural Sciences, Brussels, Belgium (RBINS).

### Position of*Tatuidris tatusia* in the food web

A nitrogen stable isotope analysis was conducted on *Tatuidris tatusia*, 20 other ant species, and other arthropods among leaf-litter mesofauna organisms (body size ranging from 0.1 to 2 mm, sensu Swift et al. 1979). Taxa used for isotopic analysis were selected on the basis of their abundance and their belonging to distinct trophic groups in order to have the largest possible range of isotopic signatures. The mesofauna was extracted by heat from 48 soil cores (5.3 cm diameter) collected inside the upper 5 cm organic layer using a modified high gradient extractor (Macfadyen 1961) for four days. Ants were extracted from 465 samples of leaf litter (total extracted area = 176.75 m²) using mini-Winkler extractors for 48 hr.

Between 1 and 31 ant workers and between 1 and 121 mesofauna individuals were pooled into tin capsules to obtain sufficient amounts of material. Samples were dried at 60°C for 24 hr, weighed, and stored in a desiccator until analysis (n = 2–5 replicates). Samples were analyzed with an elemental analyzer (NA 1500, Carlo Erba, www.carloerbareagents.com) coupled to a mass spectrometer (Finnigan MAT 251, Thermo Fisher Scientific, www.thermoscientific.com). The abundance of heavy stable isotopes (δ^15^N) was calculated as follows:

δ^15^N (‰) = (Rsample - Rstandard)/Rstandard x 1000

R_sample_ and R_standard_ represent the ^15^N/^14^N ratios corresponding to the samples and standard (atmospheric nitrogen), respectively. Acetanilide (C_8_H_9_NO, Merck, www.merckgroup.com) was used for interna calibration.

Limits between the different trophic levels were calculated in relation to the δ^15^N signature of a baseline, *Graffenrieda emarginata* (Ruiz & Pav.) Triana (Melastomataceae), one of the most frequent trees on the nutrient-poor soil in southern Ecuador (Haug et al. 2004; Illig et al. 2005). We assumed that two trophic levels were separated by a difference of ∽3.4‰ δ^15^N due to fractionation (Post 2002; Maraun et al. 2011).

### Behavioral observations and assessment of feeding habits

Extensive search in dead wood, leaf litter, and soil was carried out during both the rainy and dry seasons (2009-2011) to discover *Tatuidris* nests or live specimens. Successfully, a small colony (three workers and four gynes) of *Tatuidris tatusia* was found within the first 10 cm of a soil core and kept in captivity in a nest tube for 19 days (4-22 April 2011). To ensure sufficient air moisture inside the nest tube, water was poured in its inferior third and trapped with a cotton ball. Another cotton ball closed the tube opening. The nest was kept at ambient temperature. During their captivity, different food items (listed in the results chapter) were offered to the ants to study their feeding habits. Observations were carried out during the day under ordinary light conditions or at night using red light.

## Results and Discussion

### Species abundance

65 individuals were extracted from 79 m^**2**^of leaf litter in November 2009, and 96 individuals from 97.75 m^**2**^of leaf litter in March 2010. The average density of 1 individual/m^2^reached in March 2010 indicates that *T. tatusia* was relatively common at this locality. This result contrasts with the low abundances generally reported for the species at the local scale. For instance, the first record of the genus in Brazil was based on only two individuals (Vasconcelos and Vilhena 2003), and only a single worker was collected in French Guiana (Lacau et al. 2012). In our case, the use of the Winkler method probably facilitated the collection of this cryptic leaf-litter ant, but this alone cannot explain the relatively high abundance observed, as the method was used elsewhere with no such success. Rather, the location of our study site, at an elevation of 1000 m above sea level, may be favorable for *Tatuidris,* since Donoso (2012) suggested a preference of the genus for premontane areas at mid-level elevations (800-1200 m of altitude). In this direction, the species was only documented from three other Ecuadorian localities, where the elevation ranged from 850 to 1200 m (Vieira 2004; Donoso 2012). Further samplings at mid-elevations in Central and South America would help to identify *Tatuidris* habitat requirements.

### Morphology

**Figure 1.**
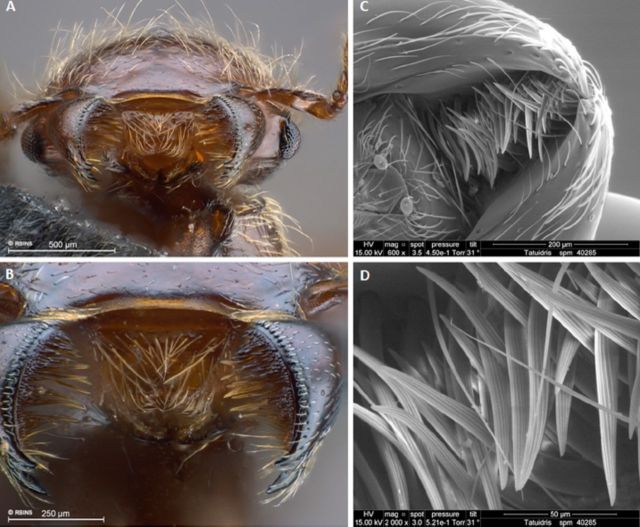
Mandibular brushes of *Tatuidris tatusia:* (A-B) anterior view [gyne, spm-ID 4657305]; (C-D) ventral view [worker, spm-ID

**Figure 2.**
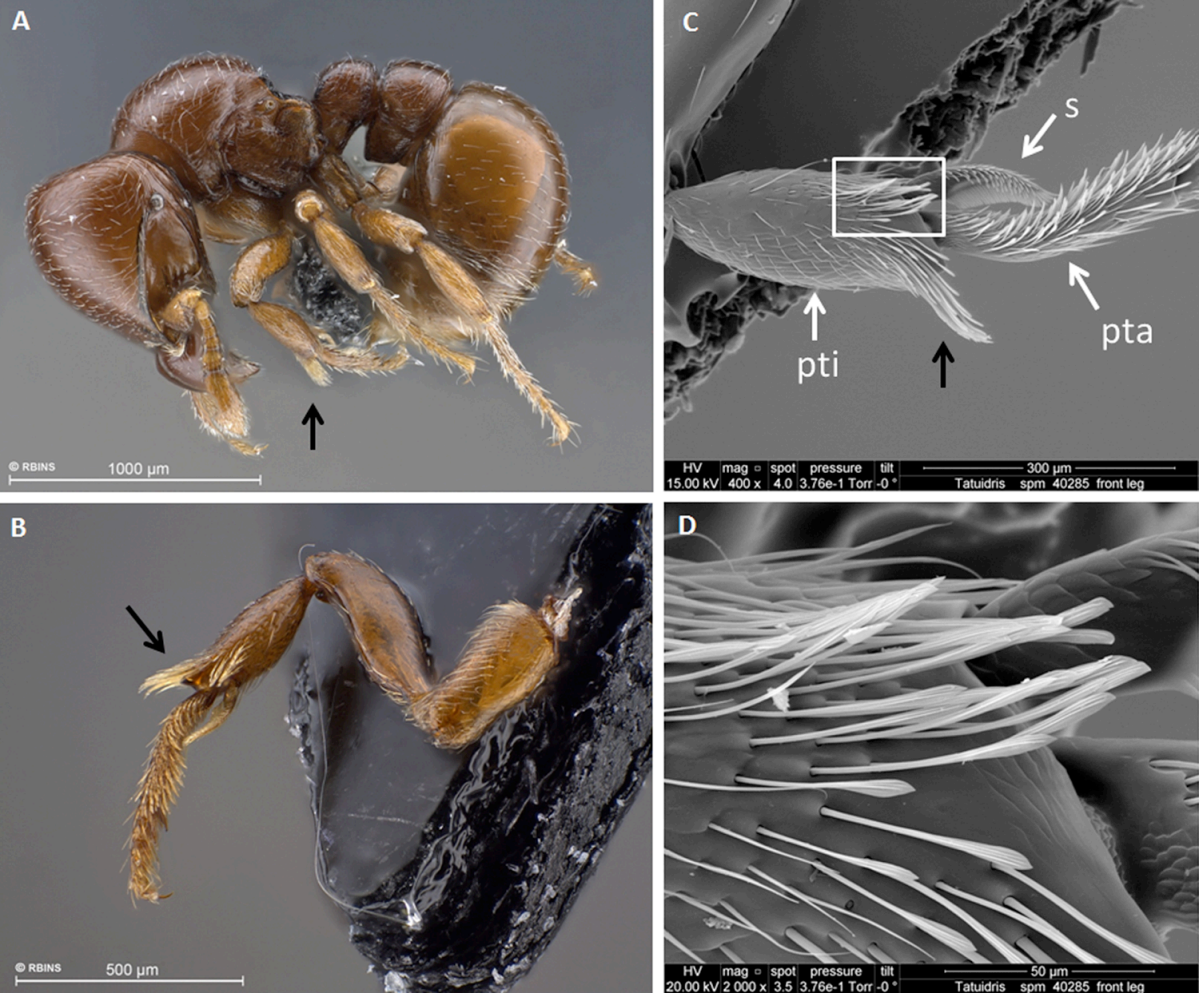
Specialized bunch of stiff setae (black arrows) on the protibia of *Tatuidris tatusia.* (A) Worker habitus, lateral view [spm-ID 33809]; (B) worker foreleg, lateroventral view; (C) worker protibia (pti), strigil (s) and upper part of protarsus (pta), lateral view of outer face; (D) detail of spatulate setae on the ventro-distal tibial angle (white rectangle on C) [B-D, spm-ID 4028512]. High quality figures are available online.

**Figure 3.**
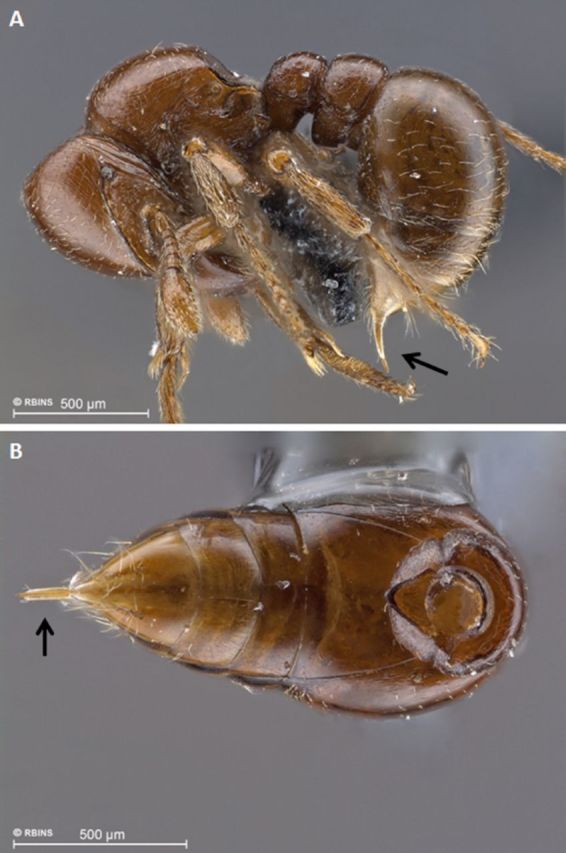
Sting of *Tatuidris tatusia* (black arrows): (A) worker habitus, lateral view [spm-ID 3431301]; (B) gaster (ventral view) and postpetiole (anterior view) [spm-ID 4028512]. High quality figures are available online.

*Tatuidris tatusia* possesses a brush of long and heavy setae along the ventral surface near the masticatory margin of the mandible (Figure 1), a bunch of stiff setae at the extensor angle on the foreleg tibia (Figure 2)—suspected to be used to clean the mandibular brush (Lacau et al. 2012)—and a strong and very long sting, relative to body size, at the apex of the gaster. The latter is projected downward and forward, which probably allows the ant to rapidly deploy its sting (Figure 3, Video 2). All these morphological peculiarities, along with round and smooth body form, suggest (as previously hypothesized) that *Tatuidris tatusia* is a specialist predator on “some active or slippery live arthropod prey” (Brown and Kempf 1968) and/or “prey bearing a defensive pilosi-ty” (Lacau et al. 2012). However, at our current level of knowledge about *T. tatusia*’s natural history, it cannot be excluded that mouthparts might be adaptations for other purposes (e.g., interactions with larvae).

### Position of*Tatuidris tatusia* in the food web

**Figure 4.**
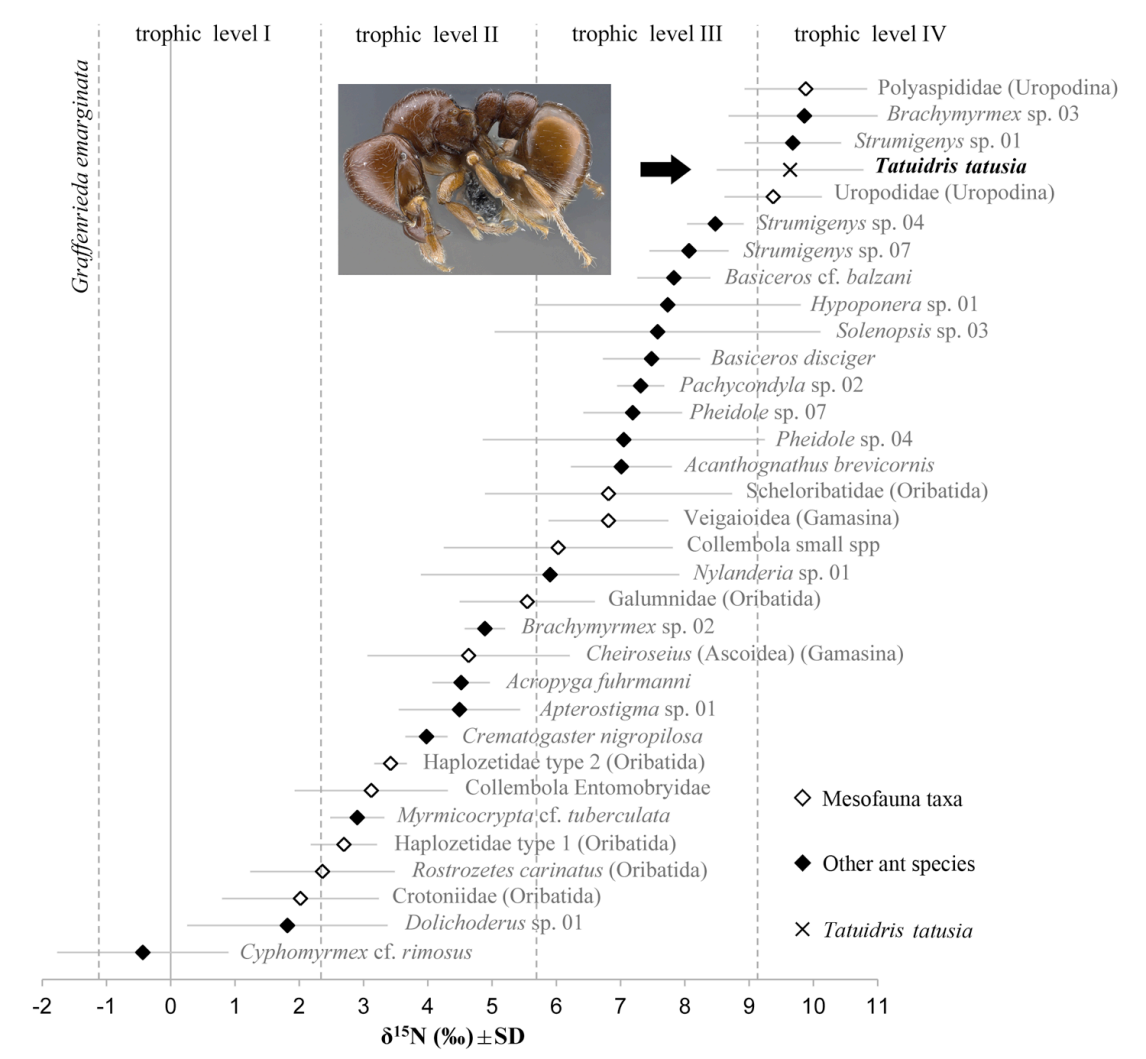
Nitrogen isotopic signatures (S^15^N, mean ± SD, n = 2-5) of *Tatuidris tatusia,* other leaf-litter dwelling ant species, and mesofauna taxa. Four trophic levels were represented. *Tatuidris tatusia* (n = 5 replicates) was in the fourth trophic level, corresponding to that of top predators. High quality figures are available online.

The average δ^15^N signature of *T. tatusia* was 9.64 ± 1.14‰ SD. The average δ^15^N signatures of the other selected taxa (other ant and mesofauna taxa) ranged between -0.43 and 9.88‰ (Figure 4). It was previously shown that these taxa belonged to the detritus-based food web (Jacquemin et al. 2012). Trophic levels were plotted relative to the δ^15^N signature of *Graffenrieda emarginata* (-1.15 ± 0.13‰ SD) as basal resource (Illig et al. 2005). Assuming that two trophic levels were separated by a difference of ∽3.4‰ δ^15^N, the gradient of 10.31 δ^15^units encompassed four trophic levels. Interestingly, *T. tatusia* was part of the fourth trophic level and was therefore one of the top predators of the leaf-litter food web under study. This result supports the hypothesis that *Tatuidris* ants are predators (Brown and Kempf 1968; Lacau et al. 2012) and suggests that *Tatuidris*’ prey is a predator itself, probably from the third trophic level. Potential predatory prey may include other ants (e.g. Dacetini) and mites (Uropodina, Gamasina) (Figure 4). *Tatuidris* could also feed on small collembolans, whose high signatures may be due to their fungal-based diet (Chahartaghi et al. 2005). Nonetheless, isotopic analysis was restricted to the most abundant mesofauna and ant taxa, and it is possible that *Tatuidris*’ prey was not included.

### Behavioral observations and assessment of feeding habits

Despite thorough and intensive search in dead wood, leaf litter, and soil during four sampling seasons at our study locality, only one small colony (three workers and four gynes) was found in a soil sample. No particular nest structure was observed, and neither brood nor food remains were present. Individuals kept in captivity did not feed on any food items offered to them, i.e. live and dead termites, oribatid mites, various insect body parts, tuna, salty biscuits, live and dead fruit flies *(Drosophila* sp.), live springtails, live myriapods (Chilopoda and Diplopoda), live and dead Diplura, small live spiders, live pseudoscorpi-ons, one small snail, ant larvae *(Gnamptogenys* sp.), and live ant workers *(Cyphomyrmex* sp., *Brachymyrmex* sp.). Similarly, cotton balls soaked with honey, sucrose dissolved in water, and fresh, whisked hen egg were not exploited by the ants, although the latter had been used with some success by Brown (1979) on *Proceratium* (see also Hölldobler and Wilson 1990). Possibly, *T. tatusia* was not interested in these food items because they were not part of its suspected specialized diet. However, we cannot reject the hypothesis that ants did not feed because they were stressed by captivity conditions.

**Video 1.** Field observation of *Tatuidris tatusia* queens (alate and dealate) and worker (6-9April 2011). Available online at: www.insectscience.org/14.108/video1.html

**Video 2.** Laboratory observation of a colony of *Tatuidris tatusia* (18 April 201 1), and observation of *T. tatusia* with other ant species *(Solenopsis* sp., *Basiceros* sp., *Strumigenys* sp., *Hypoponera* sp.) (7 December 201 2). Available online at: www.insectscience.org/14.108/video2.html

To our knowledge, this is the first time that *Tatuidris* ants were observed alive. As is observable on the videos of live specimens (Videos 1 and 2), *Tatuidris* ants moved relatively slowly. They also usually remained motionless during several tens of seconds or even several minutes when disturbed, either by our handling or by contact with other arthropods. This behavior suggests that *Tatuidris’* prey are also slow-moving animals, and that *Tatuidris* might be a sit-and-wait predator. Although not rigorously measured, ant activity seemed highest at night, suggesting that *Tatuidris* may have nocturnal habits.

Identifying ant diet is challenging. Frequently, captive ants suspected to have a specialized diet simply did not feed on offered prey or other food items, as observed, for instance, with *Probolomyrmex boliviensis* (Taylor 1965) or with *Proceratium* species (Brown 1957). Previous successful identification of specialized diet was achieved thanks to the discovery of stored food in the ant nest (e.g., arthropod eggs in nests of *Proceratium* species (Brown 1957, 1958) or Polyxenid millipedes in nests of *Probolomyrmex dam-mermani* (Ito 1998)); peculiar nesting behavior (e.g., nests of *Discothyrea oculata* in oothecas of cribellate spiders (Dejean and Dejean 1998; Dejean et al. 1999)); or prey transported by foraging workers (e.g., polyxenid millipedes by *Thaumatomyrmex* species (Brandão et al. 1991)).

Indirect prey identification through analysis of DNA fragments from gut contents (King et al. 2008; Jinbo et al. 2011) could facilitate the study of ant trophic ecology and help to understand morphological adaptations such as those exhibited by *Tatuidris* and other cryptic ant genera (e.g., *Lenomyrmex* (Fernández and Palacio 1999; Delsinne and Fernández 2012)). This approach was not attempted here because it was very likely that *Tatuidris* specimens collected with the Winkler method were contaminated by DNA from other collected organisms or regurgitated material (King et al. 2008). Keeping extracted ants alive before hand-sorting (e.g., Silva and Brandão 2010) could circumvent this issue in the future.

## Conclusion

Our results suggest that *T. tatusia* may be locally frequent in the Ecuadorian Andes. The absence of interest in food items offered during our cafeteria experiment is in agreement with the hypothesis of a specialized diet. The high position of *Tatuidris* in the leaf-litter food web, revealed by isotopic analysis, supports the current idea that *Tatuidris’* specialized morphology is related to its predatory behavior.
